# Understanding the patient journey: Barriers, facilitators, and expectations in joining a chemotherapy de-escalation trial among premenopausal patients with breast cancer

**DOI:** 10.1016/j.breast.2026.104770

**Published:** 2026-03-25

**Authors:** Petya Zyumbileva, Tanja Spanic, Emma Gillanders, Elise Martin, Marzia Zambon, Maria Connolly, Maria Dutarte, Enrico Tricanico, Carmela Caballero, Sarra El-Abed, Janet Dunn, Isabelle Merckaert, Iain R. Macpherson, Aure Vanhecke, Ines Vaz-Luis, Maria Alice Franzoi

**Affiliations:** a Université Paris-Saclay, Gustave Roussy, Inserm, CESP, F-94805, Villejuif, France; b Université Paris-Saclay, Gustave Roussy, Inserm, CESP, IHU PRISM National PRecISion Medicine Center in Oncology, F-94800, Villejuif, France; c Charité – Universitätsmedizin Berlin, Berlin, Germany; d Europa Donna-The European Breast Cancer Coalition, Milan, Italy; e Europa Donna Slovenia, Ljubljana, Slovenia; f European Patients' Academy on Therapeutic Innovation (EUPATI), Amsterdam, the Netherlands; g Breast International Group, Brussels, Belgium; h Warwick Medical School, University of Warwick, Coventry, UK; i Jules-Bordet-Institut, Brussels, Belgium; j School of Cancer Sciences, University of Glasgow, UK; k UNICANCER, Paris, France; l Interdisciplinary Department for the Organization of Patient Pathways (DIOPP), Gustave Roussy, Villejuif, France

**Keywords:** de-escalation, Patient and provider views, Hormone receptor positive breast cancer, Premenopausal, Young patients

## Abstract

**Background:**

De-escalation trials aim to balance clinical outcomes and quality of life (QoL), especially for premenopausal women with early breast cancer (BC), who often experience a greater treatment burden affecting their physical and psychosocial well-being. We sought to understand their patient journey—barriers, facilitators, and expectations—when joining de-escalation trials to inform patient-centered design and implementation.

**Methods:**

OPTIMA-YOUNG, an EU-co-funded international trial, investigates a genomic assay-guided approach to de-escalate chemotherapy in premenopausal patients with early-stage hormone receptor (HR) + BC. A Co-creation Board of patients and healthcare professionals (HCPs) ensured stakeholder involvement throughout the trial. Focus groups (FGs) explored decision-making, needs, and QoL priorities related to joining a de-escalation clinical trial. Discussions were recorded, transcribed, anonymized, and analyzed using MAXQDA software.

**Results:**

The three FGs included 20 participants (11 patients, 9 HCPs) from 14 countries. Three themes emerged: 1) emotional and cognitive responses to BC diagnosis/treatment; 2) environmental, HCP, and social influences on choices; 3) coping with side effects and QoL challenges. Barriers to joining a de-escalation trial included limited emotional support at diagnosis, cross-country variations in shared decision-making, poor communication on long-term side effects, fear of recurrence, and HCPs’ tendency to overtreat younger patients. Developing training for patients and HCPs was seen essential for improving communication, shared decision-making skills, and enhancing symptom management and QoL.

**Conclusions:**

This pre-implementation study identified factors at the patient, HCP, and system levels that, if addressed, could improve the trial experience. Insights helped refine the OPTIMA-YOUNG protocol and implementation plan and may inform future de-escalation trials.

## Highlights

• Emotional distress at diagnosis and fear of recurrence act as barriers to chemotherapy de-escalation trial participation.
• De-escalation decisions are shaped by shared decision-making variability, HCP overtreatment tendency, and family dynamics.
• Measuring and addressing endocrine therapy side effects is critical to QoL and adherence in de-escalation trials.
• HCP education and patient empowerment via self-management, digital tools and peer support can facilitate trial participation.

## 1 Introduction

Advances in early detection and treatment strategies have significantly increased breast cancer (BC) survival, with 5-year survival rates exceeding 90% [1]. Following surgery, patients diagnosed with early BC remain at risk of recurrence and so are typically offered adjuvant drug treatments such as chemotherapy and endocrine therapy to reduce this risk. However, long-term toxicities from chemotherapy and endocrine therapy continue to pose challenges, particularly impacting quality of life (QoL) and treatment adherence [[2], [3], [4]]. These effects are particularly pronounced in premenopausal women [5] who experience a range of physical (e.g., fatigue [6,7], pain [8], hot flashes [9], chemotherapy-induced menopause [10]) and psychosocial (e.g., emotional distress [[11], [12], [13]], relationship, body image [14], sexual [15,16] and fertility concerns [17,18], financial toxicities [19]) burden. Consequently, there is a growing interest in de-escalating chemotherapy in this target population to reduce the burden of toxicity while maintaining treatment efficacy.

Previous research indicates that many patients with early-stage BC wish to omit or reduce chemotherapy if safety is assured [20]. One study identified factors motivating patients to consider participation in de-escalation trials, especially their desire to avoid toxicity, minimize side effects, and enhance their QoL during and after treatment [21]. However, enrolling participants in de-escalation trials is challenging because of fear of recurrence [22] and a strong preference for aggressive treatment, especially among those who view themselves as high-risk based on tumor characteristics, family history, or race [21]. This is particularly relevant for younger patients with BC who may be at risk of being overtreated solely because of their age, without careful consideration of tumor biology and extent [23,24]. While some studies have investigated recruitment strategies and factors influencing patients' willingness to join de-escalation trials [22,25], much of the research has focused on older populations [26]. In these studies, clear communication (employing patient-centered language) about the scientific basis for de-escalation [25,27], was found to be a facilitator for informed decision making,

Despite these insights, research to address the unique challenges that young premenopausal patients considering this type of research could face remains limited. To address this gap, in the context of setting up a global clinical trial investigating a chemotherapy de-escalation approach for women with hormone receptor-positive BC [28], we conducted a pre-implementation study to explore the journey of young patients with BC when joining such trials. By examining both patient and healthcare professional (HCP) perspectives, we aimed to provide insights that could better align the trial with patient's needs and expectations.

## 2 Methods

### 2.1 Settings and participants

The current study was part of the PATH-FOR-YOUNG EU co-funded project (ID 101156800) [29] which contains OPTIMA-YOUNG, an international randomized controlled trial that investigates a genomic assay-driven approach to de-escalate chemotherapy for premenopausal patients with early-stage, hormone receptor-positive, HER2-negative BC. The trial aims to identify high-risk premenopausal patients who can safely forego chemotherapy while receiving optimal endocrine therapy. To ensure stakeholder involvement across the entire lifecycle of the trial [30], a Co-creation Board of patients and HCPs was created to ensure that the trial was aligned and responds to patients’ needs as well as to improve the experience of the patients during the implementation. The board includes individuals representing the target population or with relevant experience as care providers for the target population.

During the clinical trial protocol development phase, focus groups (FGs) were conducted to explore barriers, facilitators, and expectations when joining a de-escalation trial as well as key QoL domains that should be measured and evaluated during the trial, providing insights into the patient journey, including the emotional, physical, and psychosocial needs of premenopausal patients with BC. These results were shared with the OPTIMA-YOUNG Consortium so that trial-embedded actions could be created.

Participants were recruited through various international partner organizations, including European Patients' Academy on Therapeutic Innovation (EUPATI) fellows, Europa Donna advocates, Independent Cancer Patients' Voice (OPTIMA UK Patient and Public Involvement), and the Breast International Group Patient Partnership Initiative (BIG PPI). Patients with premenopausal BC were purposefully selected for their ability to provide meaningful insights into the patient experience. HCPs, including oncologists, surgeons, and psychologists, were recruited through partner institutions, the BIG network, and other professional and research networks associated with the OPTIMA-YOUNG Consortium. All Co-creation Board members received an informational notice before participation that outlined the project's objectives, explained the participatory research methodology, and described the potential benefits of their involvement. The recruitment criteria required participants to be ≥ 18 years old and to have conversational fluency in English to ensure active participation in discussions. Following their participation, Board members were invited to review and provide feedback on the emerging outcomes and recommendations and were subsequently informed of the final outputs, thereby contributing to participant validation and enhancing the overall credibility and confirmability of the findings [31].

### 2.2 Design and theoretical framework

The analysis of the decision-making process for premenopausal patients with BC considering participation in a de-escalation clinical trial is underpinned by an adapted model of social cognitive theory (SCT) [32]. This framework emphasizes the interplay of personal, behavioral, and environmental factors. By applying SCT, the present research explored the patient journey and identified barriers and facilitators from both patients' and physicians’ perspectives in the context of clinical trial participation. This study employed a qualitative design. The COnsolidated criteria for REporting Qualitative research (COREQ) checklist was utilized for reporting [33].

### 2.3 Data collection

FGs were conducted online with patients and HCPs in January 2025 by teleconferencing using a predefined guideline (Appendix 1). The FGs were recorded, transcribed, anonymized, and analyzed using thematic content analysis.

A brief questionnaire was sent to the patients to collect basic sociodemographic data and key information about their profiles, including age, educational level, nationality, age at BC diagnosis, and prior experience with clinical trials. HCPs provided details about their work experience, including their areas of expertise, workplaces, and years of experience. FGs were conducted separately with patients and HCPs, moderated by a medical oncologist (MAF) and sociologist (EM) with qualitative research training.

### 2.4 Data analysis

Sociodemographic data were analyzed using descriptive statistics in IBM SPSS Statistics 27. Reflexive thematic analysis was conducted and involved six stages: gaining data familiarity, inductive coding, generating initial themes, refining those themes, defining them, and naming and reporting findings [34]. MAXQDA Analytics Pro 24 facilitated the organization of codes into sub-themes and overarching themes. PZ performed open coding of the transcripts, which were then categorized into broader categories to develop the central themes. The authors (PZ and MAF) examined and refined these themes to ensure their accuracy and depth through a series of iterative analytic meetings. During these meetings, codes, candidate themes, and their interrelationships were critically examined. To enhance credibility, the resulting themes and emerging practice recommendations—supported by illustrative participant quotations—were presented and discussed within a multidisciplinary research team comprising experts in oncology, public health, and sociology (IVL, EM). These discussions facilitated further critical reflection on interpretation, contextualization, and theoretical alignment. Consistent with reflexive thematic analysis, formal intercoder reliability metrics were not calculated. Instead, analytic rigor and trustworthiness were ensured through transparent documentation, iterative team-based reflexive meetings, multidisciplinary triangulation, and the use of rich, verbatim quotations to ground interpretations in participants’ accounts.

To maintain participant confidentiality, pseudonyms were used, and personal information was anonymized.

### 2.5 Ethics

A co-design qualitative research protocol was approved by the Institutional Ethics Review Board of Gustave Roussy (N°CSET: 2024-421). No written informed consent was required; nevertheless, all participants received a participation information form explaining the study scope and detailed aspects regarding voluntary participation, data rights, and protection, and gave their verbal informed consent at the start of each FG.

## 3 Results

### 3.1 Population

**Fig. 1:**
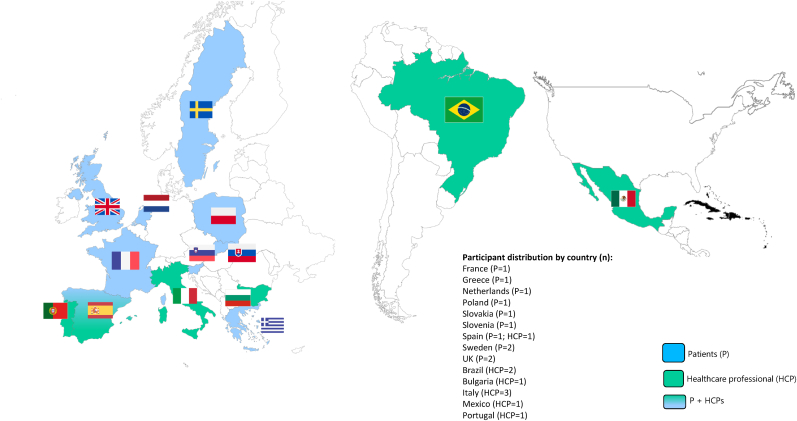
Countries represented within the PATH-FOR-YOUNG Co-creation Board.

In January 2025, three FGs were held, with 20 participants: 11 patients (two FGs) and 9 HCPs (one FG). A total of 14 countries were represented, allowing a description of the patient journey across various healthcare systems and practices (Fig. 1). All 11 patients had prior experience of BC and were diagnosed with BC before menopause.

A summary of participant characteristics is presented in Table 1.

**Table 1:** Patient and healthcare professional characteristics.

	N (%)^a^/mean (SD)
**Patient Characteristics (n = 11)**	
**Female sex**	11 (100%)
**Current Age** in years	49 (12.2)
**Age at Diagnosis** in years	38 (8.1)
**Time Since Primary Diagnosis** in years	10.9 (6.1)
**Breast Cancer Stage**	
Early stage	5 (45%)
Metastatic stage	4 (36%)
Unknown	2 (18%)
**Prior Enrolment in a Clinical Trial**	
Trial experienced	2 (18%)
Trial naïve	8 (73%)
Unknown	1 (9%)
**Country of Residence**	
France	1 (9%)
Greece	1 (9%)
Netherlands	1 (9%)
Poland	1 (9%)
Slovakia	1 (9%)
Slovenia	1 (9%)
Spain	1 (9%)
Sweden	2 (18%)
UK	2 (18%)
**Highest Level of Education**	
High School	0 (0%)
Undergraduate Degree	3 (27%)
Master's/Postgraduate/Doctorate	8 (73%)
**Healthcare Professional Characteristics (n = 9)**	
**Sex**	
Male	3 (33%)
Female	6 (67%)
**Profession of the Healthcare Professional**	
Medical oncologist	4 (44%)
Breast surgeon	3 (33%)
Breast cancer provider	1 (11%)
Psychologist	1 (11%)
**Country of Residence**	
Brazil	2 (22%)
Bulgaria	1 (11%)
Italy	3 (33%)
Mexico	1 (11%)
Portugal	1 (11%)
Spain	1 (11%)
**Professional Clinical Experience**	
<5 years	1 (11%)
5–10 years	3 (33%)
10–20 years	3 (33%)
>20 years	2 (22%)

a Due to rounding, the numbers do not always add up to 100%.

### 3.2 Emerging themes

Three major themes emerged and are shown with subthemes and exemplary quotes in Table 2: *Emotional and cognitive responses to BC diagnosis/treatment****;*** *Influence of environmental, social, and HCPs’ factors on treatment decisions****,*** and *Coping with side effects and QoL challenges.*

**Table 2:** Emergent themes and subthemes.

Themes	Subthemes	Quotes
**1. Emotional and cognitive responses to breast cancer diagnosis/treatment**	**Initial Diagnosis Shock and Difficulty Processing information**	*“So when I was diagnosed, I was completely, completely … it was completely out of the blues, something like that. And I was in shock. I didn't know what the doctors talked to me, what they are trying to tell me. I know that it was very difficult (Patient, Sweden)”*
	**Lack of Prior Knowledge about Cancer Treatments**	*“And when you are young and they told: you have a breast cancer; at that time you probably don't know anything about the disease and nothing about the treatments. So it's impossible for you at that time to decide for yourself what is good for you or what's not. (Patient, Slovenia)”*
	**Patient empowerment through addressing their information needs**	*“One of the issues nowadays still that we have is that, we say: you need to do chemotherapy, but actually we don't say: “what is your risk if you don't do chemotherapy and what is your benefit if you do chemotherapy”. And young people nowadays request this information. And I think that we as clinicians, medical doctors need to provide this data ahead so that they can do an informed decision (Psychologist, Portugal)”* *“I would need the doctor to explain why you're doing all this? What is the benefit? What's the end game? But also explain the risks of me doing or not doing the chemotherapy. So how does it change the prognosis in details? How does that affect my specific type of cancer? (Patient, Netherlands)*
**2. Influence of environmental, social, and healthcare providers' factors on treatment decisions**	**Cross-Country Differences in Resource Availability and Access to Genomic Testing and Therapy/Logistical Challenges**	*“we don't have genomic testing (…) And I think that the barriers are more related to logistic and administrative problems. But one of the things that I thought is that maybe the chemotherapy or endocrine therapy might not be available all the time. Because we have problems with that (Medical oncologist, Mexico)”*
	**Cultural Norms in Decision-Making**	*“I found out that in the Netherlands the process for treatments is … Between patients and doctors, the discussion … and there's joint decision making and this really opened the door to me to start thinking about all these. (Patient, Netherlands)* *“In Sweden, we really are … we've come quite far in shared decision making. So you could never put a treatment on a patient, said you. You will have this. You have to, you know, have a shared one (…) We know that then also the patients will be more content in the end because they have been part of this discussion. But then again we have all sorts of patients, right. We have those that do not want to take the decision. (…) So yeah, it's not easy for the doctors. They have to be very individualized and see the person, have a holistic view on what they have in front of them (Patient, Sweden)*
	**Healthcare Providers' (HCPs) Hesitation and Over-Treatment Tendencies**	*“We're, we're trained to over treat patients since we begin our training and we don't really care about over treating our patients. We really, really don't sleep well when we think we're under treating them (Medical oncologist, Brazil)”* *“it's at the end, when you're talking about something very serious that like cancer, you know, they don't want to go through the risk of, you know, having to decide alone by themselves and all those things. So this is something you know, really to work on, not only on the patients, and I really get your point, but I think you have to work on the physicians that are going to put the chart. Because there's the one that are going to pre-decide if they actually propose the trial to the patient or not. (Medical oncologist, Brazil)”*
	**Educating Physicians and the Importance of Doctor-Patient Communication**	*“it would be helpful, if the actual doctors, if the oncologists, for example, each doctor who is in this whole cancer treatment part, are actually convinced about the benefits of their patients being in a clinical trial, and in my experience, the word clinical trial itself, might be a bit scary for patients, because clinical trial, from all the decades, and from whatever we have heard in our lives, means I'm a guinea pig. (Patient, Greece)*
	**Social factors and family dynamics**	importance of the **involvement of the caregiver**/partner *“And eventually another thing to consider, let's see what the patient says, is to engage the caregiver, the partner, who is the important person for the patient. We focus too much on the patients and we forget that the other person at home is the motivator of our patients. And if that person is not in agreement with what is decided on the consultation, we may have this kind of dissonance that will make our patient uncomfortable. So it's quite important to keep it open to the partner of our patients. (Psychologist, Portugal)* *“And I think Partner Patient 1 gave a very good pass about partners and relationships and marriages, et cetera, which are I think the partner, the husband or whatever is directly affected …. is the second person after the person that is affected by all these. So I think even if this hub could be available to partners who have a section for partners or some kind of information so that they understand better the situation and what it means. Might help for a certain extent. (Patient, Netherlands)* role of **peer groups** *“I think maybe these non-profit organizations like Europa Donna and other organizations, maybe can communicate or work together with oncologists and be available for patients, to help patients with information. (Patient, Slovenia)* having **children** *“they want to be sure that they have chosen the best way to be there for the children. (Psychologist, Portugal)* *“if you have children and you get chemo … you have smaller children. They will be marked for their future because seeing their mother losing here, getting really looking different, being very tired and not being able to do things. For myself, I was admitted to hospital on Christmas Eve, so that went … my children were smaller than. And you know, it affects them still really much. I mean, a lot of other things happen in our family, but I know that this was really hard. And it is hard not on children. And if the mother doesn't have to have chemo, the children get out of that much better than if the mother has gotten chemo. (Patient, Sweden)”*
**3. Coping with Treatment Side Effects and Quality of Life Challenges**	**Managing Physical and Psychosocial Side Effects**	**Physical symptoms** (fatigue, insomnia, nausea, pain, hot flashes, etc.) *“For me, the by far the worst was the fatigue and the lack of energy and feeling like I've lost control of my body in general. Being super bloated, having stomach pains or nausea, being super tired. I think these were more … or the danger of neuropathy that comes and goes sometimes. Yeah. I think for me, these were the biggest things and also if I think … at least in my case, it was the last to go away like it lasted for months afterwards, so the most, the most scary. (Patient, Netherlands)* **Altered self-perception** *“I think during this chemo it kind of changes how who you are when you look yourself in the mirror, you don't really recognize yourself anymore(..)And it also changes you in the eyes of others (…) The fact that you lose your hair, which is, you know it. In most cases, it grows back. But for the others that look at you, you are kind of marked forever. Oh yeah, that's her. She had cancer once” (Patient, Sweden)* **Marital issues and sexual dysfunction** *“and also this the sexual dysfunction. That was a real shock, you know. I mean, I have not had penetrative intercourse since I had chemotherapy. It's just not been physically possible for me, and you know, I've got husband and you know. It's very sad so. (Patient, UK)* *“And on the part of the quality of life, these women are usually raising their families and we forgot a lot the husbands. And many of these patients, we have to understand that the outcome of their treatment is a divorce or problems in their marriage. So I think that one of the issues that we may gain the patients is that when we de-escalate the treatment, they will have less side effects and a better quality of life. And eventually they will have more possibilities to keep a safe relationship. (Psychologist, Portugal)* **Work and financial toxicities** *“And I was freelance as well when I was going through my chemotherapy. So having to work through, you know … I was sort of take a week off when I was with the side effects, we're going to be most challenging and then go in for two weeks and cram as much work as many hours in as possible so that I still had enough income to survive on. That was really hard. (Patient, UK)*
	**Tackling Endocrine Therapy Side Effects and Adherence to Endocrine Therapy**	*“And after three years, I stopped eating hormones because it was too side effects and it was so impossible to live with these side effects. So I was the one at that time who said, okay, I take my responsibility for my body (Patient, Slovenia)* *“And chemotherapy was bad but endocrine therapy was much worse. It took me … it lasted five years and the side effects were terrible and I'm still having them because especially pain. Even though I took and I still take morphine-derivated pills and also because of my experience as a patient advocate in collaborating with other working groups, there is a problem with endocrine treatments that because of side effects, there is a huge problem with the other ends. Many patients don't take the treatment because of side effects (Patient, Spain)* *“I've had some very motivated patients tell me after two or three years that they didn't want to go through a hormonal therapy anymore because it's horrible and because they want to be well, they don't want to get well, they want to be … you know, get my point? So, I think that something is very important to understand maybe when you do your quality of life research is what's really important for these women on the long run? Because we talk about hot flashes and stuff, but I think sometimes. I get an impression that ovarian suppression might be even more toxic for quality of life, at least. (Medical oncologist, Brazil)”*
	**Importance of Supportive Care Tools**	*“but there is the concept of pre-habilitation. I don't know if you have heard of it, pre- instead of rehabilitation: pre-habilitation. Which is to prepare our patients ahead of time of what will be the issues that they are to endure and how they can deal with them beforehand so as not to be surprised. And one of the things is, as I mentioned, marital issues related to sexuality. Because when we are talking about young women and endocrine therapy and suppression, we are talking about their sexuality. And if the couple is prepared to what is going to happen, that's not the, the women's fault, that's not the doctor's fault, but it's the treatment that produces this side effect and how they can deal with it. I think that we will have better outcomes in terms of quality of life and patient satisfaction than if we will not do it. So this is just a tip for what we could do in terms of supportive care for these women. Not to think just after, but a little bit ahead of time.. (Psychologist, Portugal)* *“But I also think something that is not spoken a lot about is doing psychotherapy or some kind, some form of therapy that could support the woman throughout these years. And I think this could also come as a … maybe not a recommendation by the doctor but exploring ways: If you get support, how are you coping? Because it does have a very huge mental load on the people in the long term. (Patient, Netherlands)*

#### 3.2.1 Emotional and cognitive responses to BC diagnosis/treatment

This initial theme focused on patients' emotional and cognitive reactions after receiving a BC diagnosis and making treatment choices. Within this theme, there were three subthemes: 1) Initial Diagnosis Shock and Difficulty Processing Information, 2) Lack of Prior Knowledge about Cancer Treatments, and 3) Patient Empowerment Through Addressing Their Information Needs.

Patients often experience intense shock upon diagnosis, which hinders their ability to process information, thus acting as a barrier to decision-making concerning de-escalation trials. Many participants felt overwhelmed and preferred to seek guidance from their doctors rather than engage actively in the decision-making process. Additionally, limited prior knowledge of BC and its treatment options made it challenging for them to comprehend healthcare information, resulting in reliance on physicians for treatment decisions. A substantial need for comprehensive and personalized information regarding diagnosis, treatment options, and potential outcomes emerged. Patients appreciated clear explanations and objective data from oncologists, whether delivered in paper or digitally. Furthermore, non-profit patient organizations were identified as valuable resources to provide guidance on clinical trials. Participants exhibited varying levels of willingness to accept treatment risks: some pursued all available options for reassurance, while others sought alternatives to mitigate risks. Effective communication and tailored education from HCPs were mentioned as critical facilitators that could aid patients in processing information and making informed decisions.

#### 3.2.2 Influence of environmental, HCPs’ and social factors on treatment decisions

The second theme focused on external factors, including systemic, clinical, and social influences that affect patients’ treatment decisions. Within this theme, there were six subthemes: 1) Cross-Country Differences in Resource Availability and Access to Genomic Testing and Therapy/Logistical Challenges, 2) Cultural Norms in Decision-Making, 3) HCPs' reported reluctance/tendency towards recommending more intensive therapy for younger patients, 4) Educating Physicians and the Importance of Patient-Physician Communication, and 5) Social Factors and Family Dynamics.

Significant differences in resource availability and access to genomic testing across countries were identified as structural barriers to participation in de-escalation trials. Cultural norms in decision-making varied, with Sweden emphasizing shared decision-making (SDM) in routine care, while Greece and Brazil followed a less-SDM oriented model, impacting patient autonomy. Participants noted that HCPs were often reluctant to recommend clinical trials due to perceptions of risk. This hesitation was intensified by conflicts between traditional treatments and trial options for younger patients with curable conditions, as well as HCPs' uncertainty about the safety of de-escalation trials and a trend toward accepting overtreatment. Education and HCP training emerged as key facilitators, highlighting the need for improved understanding of the scientific rationale and treatment options as well as communication skills to help patients engage in the SDM process. Social factors and family dynamics were also important, with caregivers and partners mentioned as relevant stakeholders to be welcomed into treatment discussions, if approved by the patient. Printed and digital informational materials were encouraged to facilitate SDM regarding treatment de-escalation.

#### 3.2.3 Coping with side effects and QoL challenges

The third theme focuses on behavioral factors related to perceived patient beliefs and outcome expectations, resulting in different coping strategies, management of side effects, and QoL challenges. It includes three subthemes: 1) Managing Physical and Psychosocial Side Effects, 2) Tackling Endocrine Therapy Side Effects and Adherence to Endocrine Therapy, and 3) Importance of Supportive Care Tools.

Participants reported various physical and psychosocial side effects from chemotherapy and endocrine therapy. As the care journey began, younger patients prioritized survival outcomes. However, as they experienced treatment-related side effects, many started to value QoL equally. Opinions on chemotherapy side effects varied; some acknowledged they could be intense but were typically temporary, reversible, and manageable. Fatigue was mentioned as a significant concern, along with rare but life-threatening side effects. The potential benefits of de-escalating chemotherapy were noted, particularly in reducing hospital visits. It was mentioned that this approach could particularly benefit patients from diverse socioeconomic backgrounds or those who need to continue working, such as independent professionals or family supporters. Chemotherapy was also associated with visible signs of illness, affecting emotional well-being, especially in younger children of mothers undergoing treatment. The impact of chemotherapy experience on marital status and interpersonal relationships was also brought up.

Participants noted that the most challenging aspect of the patient journey is the endocrine therapy phase due to persistent side effects. Many reported significant adverse effects, impacting adherence and motivation to continue treatment. Low outcome expectations—believing ongoing treatment offered limited benefits—along with reduced self-efficacy, contributed to non-adherence, with some patients discontinuing endocrine therapy. Their experiences with side effects were closely tied to their self-efficacy and confidence in managing treatment challenges. While some developed proactive coping strategies, others felt helpless, especially when long-term side effects were not discussed or addressed by the clinical team. This lack of preparation for enduring side effects from endocrine therapy was mentioned as a factor impacting QoL and treatment adherence.

Effective communication about endocrine therapy side effects and mitigation strategies as well as symptom management pathways proposed by HCPs during follow-up care has emerged as a factor to improve outcomes. Participants emphasized that clear communication could enhance patients' ability to manage side effects and reinforce their perception of the benefits of continuing treatment.

The participants also highlighted the value of supportive care tools that offer behavioral support through methods such as cognitive behavioral therapy, physical activity programs, and mindfulness. These tools were seen as facilitators that could increase self-efficacy, promote self-regulation, and help manage symptoms, especially during the often-overlooked phase of endocrine therapy. Digital tools were welcomed as facilitators of supportive care delivery. The concept of using digital tools as a “pre-habilitation educational platform”— since the beginning of the journey, preparing patients in advance for potential long-term side effects—was proposed to help patients cope and set realistic expectations.

## 4 Discussion

In this study, pre-implementation qualitative work within the OPTIMA-YOUNG clinical trial [29] identified modifiable barriers at patient, HCP, and system levels, which were translated into trial-embedded actions such as enhancing patient and HCP empowerment, supporting informed participation, and promoting personalized care within de-escalation chemotherapy settings across different countries and healthcare systems.

By applying the SCT framework as a theoretical guidance [32] for our analysis, we identified personal, behavioral, and environmental factors that influence patient decision-making in the context of de-escalation trials. Personal factors, such as emotional and cognitive responses upon cancer diagnosis, and behavioral factors, including patient beliefs, perceptions, and coping strategies, can hinder trial participation. In contrast, factors that may promote participation include environmental influences like HCP support and positive social support systems. These factors can be addressed as potential barriers and facilitators of trial participation, enhancing our understanding of how external and social influences, individual beliefs, knowledge, emotional capacity, and behavioral components based on perceived risks and outcome expectations shape the choices patients make regarding their involvement in clinical trials. This analysis provides actionable insights for improving patient experience and participation in future studies, such as implementing targeted educational outreach to address misconceptions about trial safety, training, and tools to encourage SDM [35].

Our research corroborates previous findings regarding the wide range of long-term treatment side effects experienced by young patients with BC [36] affecting physical and psychosocial domains [13,37,38]. Furthermore, this study aligns with previous research indicating that endocrine therapy significantly affects QoL, especially in young premenopausal women with BC, even when their treatment plans do not involve chemotherapy [39,40], as well as the impact of unaddressed symptom burden from endocrine therapy on adherence [41,42].

In this context, considering that some patients may forgo chemotherapy because endocrine therapy alone is expected to achieve similar outcomes, it is crucial to address symptom management during endocrine therapy and not neglect this aspect of the patient journey. Proactive assessment and management of endocrine therapy-related side effects may enhance QoL along with improved adherence [40]. Addressing this concern is especially important when considering the patient journey of women with BC participating in chemotherapy de-escalation trials. Employing digital tools could bridge the gap between healthcare resources and patient needs while addressing disparities in rural areas and healthcare systems with limited in-person resources [43]. Future studies should assess how these tools can enhance the QoL, assist in treatment management, and improve follow-up [44]. This support is crucial for promoting positive coping strategies and ensuring that patients feel prepared and supported throughout their entire treatment journey.

Family dynamics emerged as another significant social factor in decision-making. While previous studies have pointed out that family responsibilities, especially caring for children, can hinder participation in de-escalation trials [21,26], our research offers an additional unique perspective. Some participants saw de-escalation strategies as a means to lessen the burden of chemotherapy on their children or their relationship, illustrating that family considerations are complex and require evaluation and tailored communication. As a principle of patient-centered care, HCPs should welcome family members in discussions about treatment options and assist patients in navigating their priorities and concerns within a broader family context [45].

Our findings revealed critical systemic differences, demonstrating that less SDM-oriented healthcare systems might create significant barriers for patients seeking to participate in clinical trials. Thus, a personalized approach should be adopted, recognizing the distinct needs, outcome expectations, and preferences of each patient. SDM plays a crucial role in this process, allowing patients to actively participate in decisions about their treatment, which can facilitate trial participation, as found in previous studies [46]. HCPs play a major role in discussions about treatment options, including the potential omission of chemotherapy, which can help patients make informed choices, as described in previous research [21]. However, HCPs sometimes hesitate to recommend de-escalation trials because of concerns about risks, reflecting a broader tendency of overtreatment, even when evidence suggests a need for reduced chemotherapy intensity [20]. Therefore, HCP education regarding the scientific rationale for de-escalation seems crucial. This can enhance communication and empower HCPs to confidently present trial options, fostering a more patient-centered model of care and facilitating trial participation. Training in SDM can also be implemented; previous research shows that education on this topic is associated with increased patient satisfaction and autonomy [47], also reducing emotional distress for some patients [48]. Furthermore, previous studies have highlighted the high demand for patient decision aids to facilitate SDM and stressed their potential to improve patient satisfaction [49,50]. Similar findings about the need for tailored communication emerged from qualitative research conducted within the UK OPTIMA study [25], although this work did not focus specifically on pre-menopausal patients. The study highlighted the importance of training HCP and personalizing communication during patient recruitment. It also described the development of communication vignettes as a tool to support approaching eligible patients and encouraging their participation in de-escalation trials [25].

This study has some limitations. It focused on chemotherapy de-escalation and did not examine other de-escalation strategies, such as radiotherapy or surgical interventions, which may be associated with different perspectives. Additionally, the perspectives gathered were retrospectively reported by patients and HCPs, potentially limiting our understanding of the actual experiences of patients currently facing treatment decisions. Selection bias must be acknowledged, as recruiting through advocacy networks and requiring English fluency likely enriched the sample with highly engaged, health-literate participants, potentially overestimating empowerment and underrepresenting underserved or non-English speakers. This sampling approach may underestimate barriers experienced by patients with lower health literacy, limited digital access, socioeconomic constraints, or restricted access to specialized oncology services and genomic testing. However, we also recruited patient advocates who are trained to represent broader patient populations and who maintain active engagement with diverse networks across their native countries. Their ongoing interaction with patients across different healthcare contexts provided insights beyond individual experiences. Nevertheless, structural and contextual inequities may influence patients' understanding of trial rationale, their ability to participate in shared decision-making, and the feasibility of implementing patient-centered strategies in routine care. Therefore, there is certainly space for specific research on how to actively engage underrepresented communities in research and adopt co-design approaches to identify context-specific barriers, as well as to support culturally and linguistically adapted implementation of de-escalation trials. The strengths of this study lie in its exploration of young patients with BC's perspectives—a group that has not been systematically studied before [26]—alongside the perspectives of patients and HCPs across multiple countries, reflecting distinct cultural norms and healthcare system realities. This investigation was nested within a co-creation initiative within the protocol development and implementation planning of an ongoing clinical trial to ensure an action and solution-oriented approach.

**Fig. 2:**
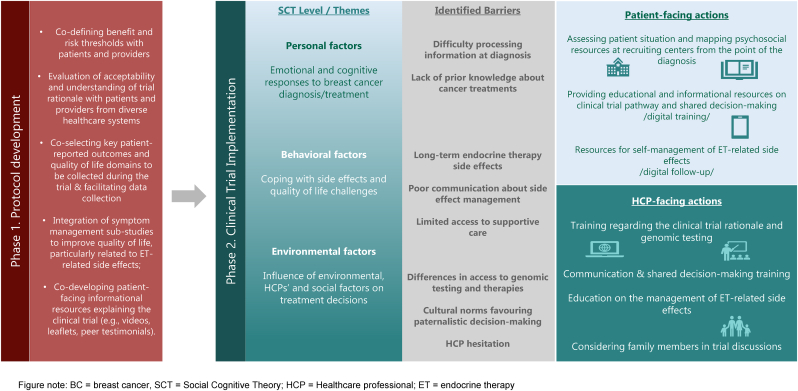
Workflow for a patient-centric approach for the implementation of a chemotherapy de-escalation clinical trial with premenopausal patients with BC.

Based on our findings, we propose a set of recommendations for improving the journey of premenopausal patients with hormone receptor-positive BC in a de-escalation chemotherapy trial (Fig. 2). To enhance implementation guidance, these strategies are presented in a structured matrix (Table 3) that both prioritizes actions according to timeframe and structural complexity—distinguishing immediate, high-impact actions from supportive medium-term initiatives and structural long-term strategies—and explicitly links identified barriers to targeted patient- and healthcare professional–focused measures across different trial phases. These recommendations are currently being implemented in the OPTIMA-YOUNG trial [29].

**Table 3:** Barrier–Action Matrix (Recommendation; Target Stakeholder; Integration and Time horizon).

Identified Barrier	Strategic Recommendation	Target Stakeholder(s)	Integration Point (Research Phase)	Time Horizon
**Difficulty processing information at diagnosis**	Patient-facing informational resources	Patient	Protocol design	Immediate action
**Lack of knowledge about treatments**	Patient-facing informational resources	Patient	Study conduct	Medium-term strategy
**ET side effects**	Symptom management sub-study + self-management resources HCP training on management of ET side effects	Patient HCP	Protocol design Study conduct	Immediate action Medium-term strategy
**Poor communication**	HCP communication training, patient empowerement information	HCP Patient	Study conduct	Immediate action
**Limited supportive care**	Supportive care referrals mapping at recruiting centers	Patient HCP	Study conduct	Medium-term strategy
**Access and availability to genomic testing**	Policy/Communication/Reimbursement routes Training on novel care pathways for system adoption	HCP	Results Dissemination	Long-term strategy
**Paternalistic norms**	Training in Shared Decision-Making	HCP	Study conduct	Immediate action
**HCP hesitation**	Trial Rational training	HCP	Study conduct	Immediate action

Legend: HCP: Healthcare professional.

ET: endocrine therapy.

During the protocol development phase, strategies include: i) co-defining benefit and risk thresholds with patients and HCPs; ii) carefully evaluating acceptability and understanding of trial rationale with patients and HCPs from diverse healthcare systems; iii) co-selecting key patient-reported outcomes and QoL domains to be collected during the trial that truly reflect the patient journey as well as facilitating data collection (readability, acceptability, prioritization, dematerialized collection); iv) integrating symptom management sub-studies to improve QoL, particularly related to endocrine therapy-related side effects; and v) co-developing patient-facing informational resources explaining the clinical trial (e.g., videos, leaflets, peer testimonials).

During the implementation phase, strategies include adopting a comprehensive approach that includes both patient and HCP-facing actions. This includes for patients: i) Assessing patient situation and mapping psychosocial resources at recruiting centers to ensure that patients are supported from the point of diagnosis and are well positioned for SDM; ii) Providing patients with educational and informational resources about the clinical trial pathway, treatment options, both short- and long-term side effects, and a SDM process. For this purpose, a digital training course, developed in partnership with patient associations, is being created for use during the trial; iii) Providing resources for the self-management of long-term endocrine therapy-related side effects – through the implementation of a nested symptom management clinical trial investigating digital tools to improve follow-up care during endocrine therapy phase, as well as symptom management strategies and mapping supportive care resources available at clinical centers participating in the trial.

Strategies for HCP-facing actions include the delivery of training resources focused on: i) The clinical trial rationale and genomic testing; ii) Communication and SDM; iii) Management of endocrine therapy-related side effects, including mapping of available supportive care strategies; iv) Considering family members and caregivers in trial discussions.

Metrics of uptake and impact of these actions will be collected during the trial and include: the reach and engagement with the SDM and empowerment resources for patients and HCPs, the overall ePRO completion in the main OPTIMA-YOUNG trial and specific efficacy/implementation endpoints for the nested clinical trial on digital symptom management support.

## 5 Conclusion

In conclusion, this pre-implementation study successfully engaged patients and HCPs in the design and implementation plan of a de-escalation trial. It identified key factors at the patient, HCP, and healthcare system levels that, if addressed, could improve the clinical trial experience. These insights helped refine the OPTIMA-YOUNG protocol and its implementation plan to better align with patient needs, and may guide future de-escalation trials among premenopausal patients with BC.

## CRediT authorship contribution statement

**Petya Zyumbileva:** Writing – review & editing, Writing – original draft, Visualization, Methodology, Formal analysis. **Tanja Spanic:** Writing – review & editing. **Emma Gillanders:** Writing – review & editing, Project administration. **Elise Martin:** Writing – review & editing, Methodology, Data curation. **Marzia Zambon:** Writing – review & editing. **Maria Connolly:** Project administration, Writing – review & editing. **Maria Dutarte:** Writing – review & editing. **Enrico Tricanico:** Writing – review & editing. **Carmela Caballero:** Writing – review & editing. **Sarra El-Abed:** Writing – review & editing. **Janet Dunn:** Writing – review & editing. **Isabelle Merckaert:** Writing – review & editing. **Iain R. Macpherson:** Writing – review & editing. **Aure Vanhecke:** Writing – review & editing, Project administration. **Ines Vaz-Luis:** Writing – review & editing, Validation, Supervision, Resources, Investigation, Conceptualization. **Maria Alice Franzoi:** Writing – review & editing, Writing – original draft, Validation, Supervision, Methodology, Investigation, Funding acquisition, Formal analysis, Data curation, Conceptualization.

## Declaration of competing interests

The authors have declared no conflicts of interest.
