# Supplementary material for: Understanding the patient journey: Barriers, facilitators, and expectations in joining a chemotherapy de-escalation trial among premenopausal patients with breast cancer
Source: Breast. 2026 Mar 25;87:104770. doi: 10.1016/j.breast.2026.104770 (PMC13091061; doi:10.1016/j.breast.2026.104770)
Supplement: Multimedia component 1 [file mmc1.docx]

**Focus Group Guide: Co-Creation Workshop with Patients and Providers - PATH FOR YOUNG Trial**

1. **Housekeeping and Overview (3 min)**
   - Briefly review the path for the Young Co-Creation Board and provide general information on how the focus group will run.
2. **Participant Introductions (15 min)**
   - Please introduce yourself:
     - Who are you?
     - Which country are you based in?
     - What is your experience with breast cancer in young women?
3. **Introduction to the PATH FOR YOUNG Project (10 min)**
   - Present a brief overview of the PATH FOR YOUNG project.
4. **Review of Anticipated Implementation Challenges for OPTIMA Young (5 min)**
   - Discuss insights from the prior UK trial and any potential challenges that may arise in the OPTIMA Young trial.
5. **Participant Interaction and Discussion (60 min)**
   - **a. Needs of Young Women with Breast Cancer (OPTIMA Young Trial)**
     - In your opinion, what are the specific needs of young women with breast cancer that should be addressed in the OPTIMA Young trial?
   - **b. Input as Patient Representative:**
     - **i.** Do you think patients would be willing/comfortable to enter this trial?
     - **ii.** What type of information and communication would patients like to receive when considering this trial?
     - **iii.** What does it represent for young women with breast cancer to enter a trial that proposes:
       - **iv.** The opportunity to spare chemotherapy if they have a low genomic risk?
       - **v.** The chance to join a digital health study to better manage the side effects of endocrine therapy?
     - **vi.** What are the main challenges young women may face during their treatment pathway (e.g., during the chemotherapy phase and after chemotherapy)?
     - **vii.** What important aspects of young women's lives might be affected by breast cancer treatment?
     - **viii.** Are there any other points you think are important to consider when developing and implementing the trial protocol?
   - **c. Input as Healthcare provider:**
     - **i.** What recruitment challenges do you anticipate if this trial opens at your site/country?
     - **ii.** Is the blinding of the randomization clear to you? Do you understand the study intervention?
     - **iii.** What about the competitive trial landscape? Would your decision to prescribe adjuvant CDK 4/6 therapy in the adjuvant setting be influenced by this trial?
     - **iv.** What can help empower patients to make a decision about joining the trial? How can we elicit patients' preferences in a practical way (e.g., moving from a paternalistic approach to an empowered, patient-centered decision-making process)?
     - **v.** How can we foster inclusion and diversity throughout the trial?
     - **vi.** What key domains should we capture both in the short-term and long-term (e.g., ePRO list)?
     - **vii.** Are there any other points you believe are critical when considering the protocol development and trial implementation?
